# Supplementary material for: Stroke in Young Adults in Spain: Epidemiology and Risk Factors by Age
Source: J Pers Med. 2023 Apr 29;13(5):768. doi: 10.3390/jpm13050768 (PMC10221310; doi:10.3390/jpm13050768)
Supplement: Supplementary file 1 [file jpm-13-00768-s001.zip › jpm-2349755-supplementary.pdf]

SUPPLEMENTARY MATERIAL

Table S1. ICD10 codes used to identify vascular risk factors and comorbidities as secondary diagnosis in each case included in the Spanish Minimum Basic Hospital Discharge Dataset.

**Condition**	**ICD10 codes (including subgroups)**
Arterial hypertension	I10
Diabetes mellitus	E10, E11
Dyslipidaemia	E78
Smoking (current or former)	Z72.0; Z87.891; F17
Cocaine	F14; T40.5- T40.5X5S
Cannabis	F1; T40.7- T40.7X5S
Enolism	F10
Obesity	E66
Migraine	G43
Hypothyroidism	E03
Hyperthyroidism	E05.5 – E05.91
Cancer	C00-C96
Ischemic heart disease	I20; I25
Atrial fibrillation	I48
Non-congenital cardiopathy (all)	I42
Dilated cardiomyopathy	I42.0
Heart failure	I13; I50
Valvulopathy (non-rheumatic)	I34; I35; I36; I37
Valvulopathy (rheumatic)	I05; I06; I07; I08; I09
PFO	Q21.1
Chronic renal failure	I12; N18
COPD	J44
OSAH	G47.3
Intracranial atherosclerosis, n (%)	I67.2
Fibromuscular dysplasia, n (%)	I77.3
Arterial dissection (intracranial)	I67.0
Arterial dissection (carotid)	I77.71
Arterial dissection (vertebral)	I77.74
Thrombophilia	D68.5-D68.9
Antiphospholipid syndrome, n (%)	D68.61; D68.62
Systemic vasculitis (all)	M31
Moyamoya disease	I67.5

Sick cell disease, n (%)	D57.0-D57.819
Fabry disease	E75.21
Endocarditis	I33
Cardiac prothesis infection	T82.7
Brain herniation, n (%)	G93.5
Seizures, n (%)	G40.909; R56.9
Acute coronary syndrome n (%)	I21
Urinary tract infection, n (%)	N39.0
Acute renal failure, n (%)	N17
Broncoaspiration	J69
Pneumonia	J18

PFO: Patent foramen oval, COPD: Chronic obstructive pulmonary disease; OSAH: Obstructive sleep apnea-hypopnea syndrome.

Table S2. Demographic and clinical characteristics by age range

Variable	**ALL ISCHEMIC STROKE PATIENTS**												
	**18-24 N=163**	**25-29 N=274**	**30-34 N=573**	**35-39 N=1230**	**40-44 N=2485**	**45-49 N=4437**	**50-54 N=7332**	**55-59 N=10846**	**60-64 N=13561**	**65-69 N=17271**	**70-74 N=21928**	**75-79 N=25155**	**>= 80 N=81232**
Demographic dat vascular risk factors and comorbidities													
Male, n (%)	81 (49.7%)	134 (48.9%)	300 (52.4%)	773 (62.8%)	1593 (64.1%)	2993 (67.5%)	5290 (72.1%)	8014 (73.9%)	9699 (71.5%)	11556 (66.9%)	13332 (60.8%)	13607 (54.1%)	32036 (39.4%)
Arterial hypertens (%)	8 (4.9%)	16 (5.8%	55 (9.6%)	216 (17.6%	695 (28%	1746 (39.4	3481 (47.	5623 (51.8	7482 (55.2	10064 (58.3	13110 (59.	15006 (59.	45461 (56%)
Diabetes mellitus,	2 (1.2%)	3 (1.1%)	14 (2.4%)	58 (4.7%)	222 (8.9%	665 (15%)	1530 (15	3003 (27.7	4364 (32.2	6048 (35%)	8025 (36.6	9013 (35.8	24610 (30.3%)
Dyslipidaemia, n (	4 (2.5%)	21 (7.7%	69 (12%)	251 (20.4%	627 (25.2	1445 (32.6	2918 (39.	4704 (43.4	6117 (45.1	8150 (47.2%	10319 (47.	11457 (45.	30054 (37%)
Smoking, n (%)	43 (26.4%)	103 (37.6%)	239 (41.7%)	543 (44.1%)	1151 (46.3%)	2329 (52.2%)	4262 (58.1%)	6206 (57.2%)	6770 (49.9%)	6865 (39.7%)	6538 (29.8%)	5251 (20.9%)	8157 (10%)
Cocaine, n (%)	10 (6.1%	24 (8.8%	45 (7.9%)	85 (6.9%)	134 (5.4%	183 (4.1%	170 (2.3%	112 (1%)	56 (0.4%)	36 (0.2%)	7 (0%)	3 (0%)	0 (0%)
Cannabis, n (%)	14 (8.6%	33 (12%)	45 (7.9%)	84 (6.8%)	98 (3.9%	124 (2.8%	150 (2%)	122 (1.1%	54 (0.4%)	34 (0.2%)	9 (0%)	2 (0%)	3 (0%)
Enolism, n (%)	4 (2.5%)	12 (4.4%	29 (5.1%)	80 (6.5%)	241 (9.7%	498 (11.2%	1074 (14.	1738 (16%	1945 (14.3	2021 (11.7%	1676 (7.6%	1207 (4.8%	1248 (1.5%)
Cancer diagnosis,	0 (0%)	3 (1.1%)	3 (0.5%)	18 (1.5%)	38 (1.5%	81 (1.8%)	195 (2.7%	424 (3.9%	659 (4.9%	872 (5%)	1197 (5.5%	1318 (5.2%	3623 (4.5%)
Obesity, n (%)	12 (7.4%	16 (5.8%	32 (5.6%)	131 (10.7%	290 (11.7	626 (14.1%	951 (13%	1325 (12.2	1491 (11%	1944 (11.3%	2037 (9.3%	2003 (8%)	4002 (4.9%)
Ischemic heart dis (%)	4 (2.5%)	11 (4%	18 (3.1%)	51 (4.1%)	124 (5%)	250 (5.6%	484 (6.6%	903 (8.3%	1258 (9.3%	1849 (10.7%	2523 (11.5	3009 (12%	9403 (11.6%)
Atrial fibrillation,	2 (1.2%)	3 (1.1%)	12 (2.1%)	24 (2%)	104 (4.2%)	201 (4.5%)	420 (5.7%)	997 (9.2%)	1910 (14.1%)	3320 (19.2%)	5543 (25.3%)	7912 (31.5)	33977 (41.8%)
Non-congenital cardiomyopathy, r	1 (0.6%)	10 (3.6%	17 (3%)	34 (2.8%)	51 (2.1%	127 (2.9%	205 (2.8%	307 (2.8%	334 (2.5%	452 (2.6%)	492 (2.2%	549 (2.2%	1339 (1.6%)
Dilated cardiomyo n (%)	0 (0%)	4 (1.5%)	7 (1.2%)	15 (1.2%)	26 (1%)	62 (1.4%)	110 (1.5%	151 (1.4%	172 (1.3%	227 (1.3%)	233 (1.1%	278 (1.1%	598 (0.7%)
Heart failure, n (%	0 (0%)	0 (0%)	7 (1.2%	9 (0.7%	26 (1%	61 (1.4%	121 (1.7%	240 (2.2%	360 (2.7%	503 (2.9%)	835 (3.8%	1265 (5%)	7397 (9.1%)
Valvulopathy (no rheumatic), n (%)	1 (0.6%)	1 (0.4%)	6 (1%)	10 (0.8%)	19 (0.8%	50 (1.1%)	59 (0.8%	122 (1.1%	189 (1.4%	400 (2.3%)	644 (2.9%	930 (3.7%	3789 (4.7%)
Valvulopathy (rheumatic), n (%	0 (0%)	3 (1.1%)	7 (1.2%)	18 (1.5%)	32 (1.3%	44 (1%)	86 (1.2%	148 (1.4%	249 (1.8%	395 (2.3%)	567 (2.6%	763 (3%)	2834 (3.5%)
PFO, n (%)	41 (25.2%	60 (21.9%	96 (16.8%	167 (13.6%	268 (10.8	312 (7%)	292 (4%)	271 (2.5%	150 (1.1%	185 (1.1%)	146 (0.7%	109 (0.4%	127 (0.2%)
OSA, n (%)	1 (0.6%)	0 (0%)	10 (1.7%)	30 (1.7%)	95 (3.8%	230 (5.2%	428 (5.8%	693 (6.4%	863 (6.4%	1160 (6.7%	1365 (6.2%	1226 (4.9%	1861 (2.3%)
Intracranial atherosclerosis, n	1 (0.6%)	2 (0.7%)	3 (0.5%)	11 (0.9%)	46 (1.9%	92 (2.1%)	274 (3.7%	448 (4.1%	588 (4.3%	734 (4.2%)	964 (4.4%	1083 (4.3%	3074 (3.8%)
Unusual causes													
Fibromuscular dysplasia, n (%)	2 (1.2%)	0 (0%)	0 (0%)	0 (0%)	2 (0.2%)	5 (0.2%)	6 (0.1%)	5 (0.1%)	5 (0%)	0 (0%)	3 (0%)	3 (0%)	3 (0%)
Arterial dissection (all), n (%)	10 (6.1%)	10 (3.6%)	29 (5.1%)	90 (7.3%)	118 (4.7%)	144 (3.2%)	176 (2.4%)	107 (1%)	89 (0.7%)	62 (0.4%)	53 (0.2%)	44 (0.2%)	56 (0.1%
Arterial dissection intracranial), n (%)	1 (0.6%)	1 (0.4%)	3 (0.5%)	13 (1.1%)	9 (0.4%)	5 (0.1%)	10 (0.1%)	7 (0.1%)	9 (0.1%)	4 (0%)	4 (0%)	4 (0%)	7 (0%)
Arterial dissection (carotid), n (%)	5 (3.1%)	4 (1.5%)	10 (1.7%)	42 (3.4%)	60 (2.4%)	101 (2.3%)	130 (1.8%)	71 (0.7%)	63 (0.5%)	44 (0.3%)	31 (0.1%)	25 (0.1%)	33 (0%)
Arterial dissection (vertebral), n (%)	5 (3.1%)	5 (1.8%)	18 (3.1%)	35 (2.8%)	50 (2%)	38 (0.9%)	40 (0.5%)	29 (0.3%)	19 (0.1%)	14 (0.1%)	18 (0.1%)	15 (0.1%)	16 (0%)
Thrombophilia, n (%)	12 (7.4%)	14 (5.1%)	23 (4%)	27 (2.2%)	76 (3.1%)	109 (2.5%)	117 (1.6%)	137 (1.3%)	114 (0.8%)	111 (0.6%)	98 (0.4%)	90 (0.4%)	183 (0.2%)
Vasospasm, n (%)	1 (0.6%)	3 (1.1%)	3 (0.5%)	1 (0.1%)	5 (0.2%)	7 (0.2%)	9 (0.1%)	12 (0.1%)	8 (0.1%)	9 (0.1%)	3 (0%)	13 (0.1%)	14 (0%)
Arteritis, n (%)	0 (0%)	1 (0.4%)	2 (0.3%)	2 (0.2%)	4 (0.2%)	4 (0.1%)	6 (0.1%)	9 (0.1%)	7 (0%)	7 (0%)	5 (0%)	10 (0%)	15 (0%)
Systemic lupus erythematosus , n (%)	2 (1.2%)	2 (0.7%)	8 (1.4%)	9 (0.7%)	20 (0.8%)	24 (0.5%)	13 (0.2%)	28 (0.3%)	25 (0.2%)	22 (0.1%)	24 (0.1%)	19 (0.1%)	31 (0%)
Endocarditis, n (%)	0 (0%)	1 (0.4%)	1 (0.2%)	1 (0.1%)	9 (0.4%)	4 (0.1%)	8 (0.1%)	18 (0.2%)	20 (0.1%)	22 (0.1%)	27 (0.1%)	32 (0.1%)	61 (0.1%)
**Outcome**													
In-hospital mortality, n (%)	3 (1.8%)	4 (1.5%)	14 (2.4%)	31 (2.5%)	77 (3.1%)	124 (2.8%)	278 (3.8%)	498 (4.6%)	770 (5.7%)	1071 (6.2%)	1766 (8.1%)	2517 (10%)	16410 (20.2%)

PFO: Patent foramen oval, COPD: Chronic obstructive pulmonary disease; OSAH: Obstructive sleep apnea-hypopnea syndrome.

Table S3. Demographic and clinical characteristics of the included patients by sex and age group.

Variable	All patients			18-50 y.o.			> 50 y.o.		
	**Men (N=99408)**	**Women (n=87079)**	**p**	**Men (N=5874)**	**Women (n=3288)**	**p**	**Men (N=)**	**Women (n=)**	**p**
Demographic data, vascular risk factors and comorbiditi									
Mean age (SD), years	71.6 (13.1)	78.4 (12.8)	< 0.001	43.03 (5.79	41.7 (6.74)	0.000	73.36 (11.2	79.79 (10.6	< 0.001
Arterial hypertension, n (%)	53540 (53.9	49423 (56.8	< 0.001	1941 (33%	795 (24.2%	0.000	51599 (55.2	48628 (58%	< 0.001
Diabetes mellitus, n (%)	31971 (32.2	25586 (29.4	< 0.001	707 (12%)	257 (7.8%	0.000	31264 (33.4	25329 (30.2	< 0.001
Dyslipidaemia, n (%)	40898 (41.1	35238 (40.5	0.003	1772 (30.2	645 (19.6%	0.000	39126 (41.8	34593 (41.3	0.02
Smoking, n (%)	39953 (40.2	8504 (9.8%)	< 0.001	3016 (51.3	1392 (42.3	0.000	36937 (39.5	7112 (8.5%	< 0.001
Cannabis, n (%)	683 (0.7%)	89 (0.1%)	< 0.001	334 (5.7%)	64 (1.9%)	0.000	349 (0.4%)	25 (0%)	< 0.001
Cocaine, n (%)	764 (0.8%)	101 (0.1%)	< 0.001	411 (7%)	70 (2.1%)	0.000	353 (0.4%)	31 (0%)	< 0.001
Enolism, n (%)	10806 (10.9	967 (1.1%)	< 0.001	763 (13%)	101 (3.1%	0.000	10043 (10.7	866 (1%)	< 0.001
Hypothyroidism, n (%)	2417 (2.4%)	7814 (9%)	< 0.001	100 (1.7%)	229 (7%)	0.000	2317 (2.5%	7585 (9.1%	< 0.001
Hyperthyroidism, n (%)	463 (0.5%)	1053 (1.2%)	< 0.001	29 (0.5%)	34 (1%)	0.003	434 (0.5%)	1019 (1.2%	< 0.001
Cancer diagnosis, n (%)	5393 (5.4%)	3038 (3.5%)	< 0.001	65 (1.1%)	78 (2.4%)	0.000	5328 (5.7%	2960 (3.5%	< 0.001
Obesity, n (%)	7167 (7.2%)	7693 (8.8%)	< 0.001	672 (11.4%	435 (13.2%	0.012	6495 (6.9%	7258 (8.7%	< 0.001
Ischemic heart disease, n (%)	13159 (13.2	6728 (7.7%)	< 0.001	316 (5.4%)	142 (4.3%	0.025	12843 (13.7	6586 (7.9%	< 0.001
Atrial fibrillation, n (%)	23869 (24%	30556 (35.1	< 0.001	252 (4.3%)	95 (2.9%)	0.001	23618 (25.3	30461 (36.4	< 0.001
Non-congenic cardiomyopatl (%)	2622 (2.6%)	1296 (1.5%)	< 0.001	185 (3.1%)	55 (1.7%)	0.000	2437 (2.6%	1241 (1.5%	< 0.001
Dilated cardiomyopathy, n (%	1411 (1.4%)	472 (0.5%)	< 0.001	96 (1.6%)	18 (0.5%)	0.000	1315 (1.4%	454 (0.5%)	< 0.001
Enolic cardiomyopathy, n (%)	153 (0.2%)	7 (0%)	< 0.001	14 (0.2%)	0 (0%)	0.005	139 (0.1%)	7 (0%)	< 0.001
Heart failure, n (%)	4413 (4.4%)	6411 (7.4%)	< 0.001	74 (1.3%)	29 (0.9%)	0.10	4339 (4.6%	6382 (7.6%	< 0.001
Valvulopathy (all), n (%)	4726 (4.8%)	6233 (7.2%)	< 0.001	101 (1.7%)	84 (2.6%)	0.006	4625 (4.9%	6149 (7.3%	< 0.001
Valvulopathy (non-rheumatic (%)	3055 (3.1%)	3165 (3.6%)	< 0.001	52 (0.9%)	35 (1.1%)	0.39	3003 (3.2%	3130 (3.7%	< 0.001
Valvulopathy (rheumatic), n	1823 (1.8%)	3323 (3.8%)	< 0.001	51 (0.9%)	53 (1.6%)	0.001	1772 (1.9%	3270 (3.9%	< 0.001
PFO, n (%)	1293 (1.3%)	931 (1.1%)	< 0.001	562 (9.6%)	382 (11.6%	0.002	731 (0.8%)	549 (0.7%)	0.002
Chronic renal failure, n (%)	10219 (10.3	10002 (11.5	< 0.001	156 (2.7%)	52 (1.6%)	0.001	10063 (10.8	9950 (11.9	< 0.001
COPD, n (%)	8629 (8.7%)	1926 (2.2%)	< 0.001	36 (0.6%)	19 (0.6%)	0.835	8593 (9.2%	1907 (2.3%	< 0.001
OSA, n (%)	5873 (5.9%)	2089 (2.4%)	< 0.001	318 (5.4%)	48 (1.5%)	0.000	5555 (5.9%	2041 (2.4%	< 0.001
Intracranial atherosclerosis, r	4216 (4.2%)	3104 (3.6%)	< 0.001	122 (2.1%)	33 (1%)	0.000	4094 (4.4%	3071 (3.7%	< 0.001
Unusual causes									
Fibromuscular dysplasia, n (%)	14 (0%)	22 (0%)	0.08	6 (0.1%)	8 (0.2%)	0.097	8 (0%)	14 (0%)	0.124
Arterial dissection (all), n (%)	706 (0.7%)	282 (0.3%)	<0.001	279 (4.7%)	122 (3.7%)	0.020	427 (0.5%)	160 (0.2%)	< 0.001
Arterial dissection intracranial), n (%)	48 (0%)	29 (0%)	0.11	23 (0.4%)	9 (0.3%)	0.359	25 (0%)	20 (0%)	0.706
Arterial dissection (carotid), n (%)	441 (0.4%)	178 (0.2%)	<0.001	148 (2.5%)	74 (2.3%)	0.422	293 (0.3%)	104 (0.1%)	< 0.001
Arterial dissection (vertebral), n (%)	223 (0.2%)	79 (0.1%)	<0.001	110 (1.9%)	41 (1.2%)	0.024	113 (0.1%)	38 (0%)	< 0.001
Thrombophilia, n (%)	603 (0.6%)	508 (0.6%)	0.516	127 (2.2%)	134 (4.1%)	0.000	476 (0.5%)	374 (0.4%)	0.057
Vasospasm, n (%)	42 (0%)	46 (0.1%)	0.29	7 (0.1%)	13 (0.4%)	0.007	35 (0%)	33 (0%)	0.833
Systemic vasculitis (all), n (%)	157 (0.2%)	201 (0.2%)	<0.001	11 (0.2%)	14 (0.4%)	0.036	146 (0.2%)	187 (0.2%)	0.001
Moyamoya disease, n (%)	15 (0%)	17 (0%)	0.466	4 (0.1%)	9 (0.3%)	0.012	11 (0%)	8 (0%)	0.653
Sick cell disease, n (%)	6 (0%)	11 (0%)	0.137	2 (0%)	6 (0.2%)	0.021	4 (0%)	5 (0%)	0.618
Endocarditis, n (%)	116 (0.1%)	88 (0.1%)	0.308	10 (0.2%)	6 (0.2%)	0.893	106 (0.1%)	82 (0.1%)	0.318
Cardiac prothesis infection, n (%)	178 (0.2%)	157 (0.2%)	0.950	9 (0.2%)	15 (0.5%)	0.006	169 (0.2%)	142 (0.2%)	0.573
In-hospital complications									
Brain herniation, n (%)	494 (0.5%)	559 (0.6%)	<0.001	49 (0.8%)	38 (1.2%)	0.128	445 (0.5%)	521 (0.6%)	< 0.001
Seizures, n (%)	546 (0.5%)	641 (0.7%)	<0.001	25 (0.4%)	21 (0.6%)	0.166	521 (0.6%)	620 (0.7%)	< 0.001
Acute coronary syndrome n (%)	428 (0.4%)	351 (0.4%)	0.359	14 (0.2%)	12 (0.4%)	0.274	414 (0.4%)	339 (0.4%)	0.219
Urinary tract infection, n (%)	4124 (4.1%)	9474 (10.9%)	<0.001	89 (1.5%)	118 (3.6%)	0.000	4035 (4.3%)	9356 (11.2%)	< 0.001
Acute renal failure, n (%)	3524 (3.5%)	3210 (3.7%)	0.103	75 (1.3%)	20 (0.6%)	0.002	3449 (3.7%)	3190 (3.8%)	0.185
Broncoaspiration, n (%)	3880 (3.9%)	3813 (4.4%)	<0.001	63 (1.1%)	21 (0.6%)	0.037	3817 (4.1%)	3792 (4.5%)	< 0.001

Pneumonia, n (%)	1387 (1.4%)	1112 (1.3%)	0.027	25 (0.4%)	9 (0.3%)	0.251	1362 (1.5%)	1103 (1.3%)	0.012
Outcome									
In-hospital mortality, n (%)	10133 (10.2%)	13430 (15.4%)	<0.001	168 (2.9%)	85 (2.6%)	0.441	9965 (10.7%)	13345 (15.9%)	<0.001

PFO: Patent foramen oval, COPD: Chronic obstructive pulmonary disease; OSAH: Obstructive sleep apnea-hypopnea syndrome.

Table S4. Prevalence of vascular risk factors in general population by sex and age group (health survey 2014).

	≥ 18 (all)			18-50 (young)			> 50 (old)		
Comorbidity, n (%)	All (22321)	Men (10297)	Women (12024)	All (10669)	Men (5224)	Women (5445)	All (11652)	Men (5073)	Women (6579)
HBP	6023 (27%)	2675 (26%)	3348 (27.8%)	911 (8.5%)	514 (9.8%)	397 (7.3%)	5112 (43.9%)	2161 (42.6%)	2951 (44.9%)
Dyslipidemia	5166 (23.2%)	2310 (22.5%)	2856 (23.8%)	1026 (9.6%)	650 (12.5%)	376 (6.9%)	4140 (35.6%)	1660 (32.9%)	2480 (37.8%)
Diabetes Mellitus	1956 (8.8%)	942 (9.2%)	1014 (8.4%)	191 (1.8%)	103 (2%)	88 (1.6%)	1765 (15.2%)	839 (16.6%)	926 (14.1%)
Smoking	4787 (21.4%)	2629 (25.5%)	2158 (17.9%)	3096 (29%)	1705 (32.6%)	1391 (25.5%)	1691 (14.5%)	924 (18.2%)	767 (11.7%)
Excesive enolic intake	8195 (36.7%)	5304 (51.5%)	2891 (24%)	3891 (36.5%)	2470 (47.3%)	1421 (26.1%)	4304 (36.9%)	2834 (55.9%)	1470 (22.3%)
Obesity (BMI > 30)	2794 (13.7%)	1299 (13.6%)	1495 (13.8%)	943 (9.3%)	516 (10.4%)	427 (8.3%)	1851 (18%)	783 (17.1%)	1068 (18.7%)
Ischemic heart disease	929 (4.2%)	580 (5.6%)	349 (2.9%)	60 (0.6%)	44 (0.8%)	16 (0.3%)	869 (7.5%)	536 (10.6%)	333 (5.1%)
